# Stemness‐related genes revealed by single‐cell profiling of naïve and stimulated human CD34^+^ cells from CB and mPB

**DOI:** 10.1002/ctm2.1175

**Published:** 2023-01-22

**Authors:** Guoyi Dong, Xiaojing Xu, Yue Li, Wenjie Ouyang, Weihua Zhao, Ying Gu, Jie Li, Tianbin Liu, Xinru Zeng, Huilin Zou, Shuguang Wang, Yue Chen, Sixi Liu, Hai‐Xi Sun, Chao Liu

**Affiliations:** ^1^ College of Life Sciences University of Chinese Academy of Sciences Beijing 100049 China; ^2^ China National GeneBank BGI‐Shenzhen Shenzhen 518120 China; ^3^ BGI‐Shenzhen Shenzhen 518083 China; ^4^ Department of Hematology and Oncology Shenzhen Children's Hospital Shenzhen China; ^5^ Shenzhen Second People's Hospital First Affiliated Hospital of Shenzhen University Shenzhen China; ^6^ BGI‐Beijing Beijing 102601 China

**Keywords:** cord blood, human hematopoietic stem cells, mobilised peripheral blood, single‐cell RNA‐seq, stemness‐related genes

## Abstract

**Background:**

Hematopoietic stem cells (HSCs) from different sources show varied repopulating capacity, and HSCs lose their stemness after long‐time ex vivo culture. A deep understanding of these phenomena may provide helpful insights for HSCs.

**Methods:**

Here, we applied single‐cell RNA‐seq (scRNA‐seq) to analyse the naïve and stimulated human CD34^+^ cells from cord blood (CB) and mobilised peripheral blood (mPB).

**Results:**

We collected over 16 000 high‐quality single‐cell data to construct a comprehensive inference map and characterised the HSCs under a quiescent state on the hierarchy top. Then, we compared HSCs in CB with those in mPB and HSCs of naïve samples to those of cultured samples, and identified stemness‐related genes (SRGs) associated with cell source (CS‐SRGs) and culture time (CT‐SRGs), respectively. Interestingly, CS‐SRGs and CT‐SRGs share genes enriched in the signalling pathways such as mRNA catabolic process, translational initiation, ribonucleoprotein complex biogenesis and cotranslational protein targeting to membrane, suggesting dynamic protein translation and processing may be a common requirement for stemness maintenance. Meanwhile, CT‐SRGs are enriched in pathways involved in glucocorticoid and corticosteroid response that affect HSCs homing and engraftment. In contrast, CS‐SRGs specifically contain genes related to purine and ATP metabolic process, which is crucial for HSC homeostasis in the stress settings. Particularly, when CT‐SRGs are used as reference genes for the construction of the development trajectory of CD34^+^ cells, lymphoid and myeloid lineages are clearly separated after HSCs/MPPs. Finally, we presented an application through a small‐scale drug screening using Connectivity Map (CMap) against CT‐SRGs. A small molecule, cucurbitacin I, was found to efficiently expand HSCs ex vivo while maintaining its stemness.

**Conclusions:**

Our findings provide new perspectives for understanding HSCs, and the strategy to identify candidate molecules through SRGs may be applicable to study other stem cells.

## Graphical abstract

Stemness‐related genes (SRGs) associated with culture time (CT‐SRGs) and cell source (CS‐SRGs) are characterised. CT‐SRGs and CS‐SRGs share signalling pathways while show distinct gene patterns; CT‐SRGs are better reference genes for trajectory construction of CD34^+^ cell. Cucurbitacin I can efficiently expand both CB and mPB HSCs ex vivo.

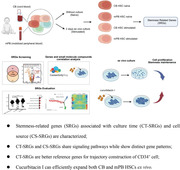

## 1 INTRODUCTION

Hematopoietic stem cells (HSCs) are responsible for initiating hematopoiesis and maintaining the homeostasis of the hematopoietic system.^1^ HSCs are defined as cells on top of the hematopoietic hierarchy with totipotency.^2^, ^3^ The molecular signatures and signalling pathways of human HSCs have been extensively investigated^4^, ^5^ and strategies to modulate those factors or signalling pathways have been explored to expand and maintain the functional HSC*s* ex vivo.^6^ Several candidates capable of efficiently expanding HSCs, including SR1 and UM171, have been identified and validated in animal models.^7^, ^8^, ^9^ However, the strategies and small molecules mentioned above have not been successfully applied under clinical settings. Thus, there are other important underlying mechanisms required for HSC maintenance to be identified.

In human, CD34^+^ cells isolated with magnetic beads containing the HSCs are clinically used for HSC transplantation (HSCT) and HSC gene therapy (HSC‐GT). On one aspect, it is well known that CD34^+^ cells derived from cord blood (CB) have stronger regeneration capacities than that from mobilised peripheral blood (mPB).^10^ In clinical practice, the minimal number of mPB CD34^+^ cells required for allogenic HSCT is usually above 2 × 10^6^/kg.^11^, ^12^, ^13^ By contrast for CB CD34^+^ cells, a quarter population (0.5 × 10^6^/kg) is sufficient for allogeneic cell transplantation.^14^ In addition, when generating humanised mice model, the required human CD34^+^ cells are 0.5 × 10^5^ of CB or 1.0 × 10^6^ of mPB cells per mouse,^15^, ^16^ further confirming the higher stemness of CD34^+^ cells from CB. Although the reasons for these biological differences are still not fully clear, several mechanisms that have been implicated, which were related to telomere dynamics, cell cycle progression, certain transcription factor pathways, differential gene expression and the autocrine production of particular cytokines.^17^ On another aspect, it is critical to keep the manipulation time of HSCs short during a successful HSC‐GT, as the capacity of HSCs to rebuild the hematopoiesis is decreased along with the ex vivo culture time.^18^ However, the differences of CD34^+^ cells from CB and mPB sources, as well as from naive and cultured conditions, are not intensively investigated. Therefore, a systematic analysis would be helpful to understand the underlying mechanism, and findings from these studies should shed light on the signature genes and signalling pathways related to the stemness of HSCs.

Increasing evidence shows that CD34^+^ cells are a heterogeneous cell population at different levels.^19^, ^20^ To fully explore the HSC complexity, single‐cell RNA sequencing (scRNA‐seq) has been intensively used in this field with two approaches: one approach is to isolate the HSCs and other progenitor cells using fluorescence‐assisted cell sorting (FACS) with different antibodies against cell surface markers, and then perform the scRNA‐seq in those “characterised” cell populations, respectively^21^; another approach is to perform scRNA‐seq on unsorted CD34^+^ cells and then define the sub‐cell populations based on bioinformatic analysis.^22^ However, as the first approach still relies on the expression of surface markers, which may introduce bias in cell definition, it may not fully cover and reveal the bona fide cell populations as considerable cells are discarded during FACS process. Moreover, recent studies have found HSCs may also exist in other cell populations, suggesting the population characterisation based on cell surface markers are not sufficient.^23^ By contrast, the second approach, in theory, is able to capture all kinds of cells in an unbiased way during hematopoiesis, by which cell populations are clustered and defined by their gene expression profiles.^21^, ^24^, ^25^ scRNA‐seq of unsorted CD34^+^ cells has revealed hierarchically structured transcriptional landscape of hematopoietic differentiation and has identified new markers of HSCs and other progenitor cells. However, no systematic analysis has been performed to understand the differences between CD34^+^ from CB and mPB or between naïve or stimulated CD34^+^ cells.

Here, we collected CB and mPB CD34^+^ cells from independent individuals, conducted the scRNA‐seq immediately or after 2 days ex vivo culture. With the comprehensive scRNA‐seq dataset, we performed bioinformatic analysis to characterised HSC population and identified two sets of stemness‐related genes (SRGs) associated with culture time and cell source, termed as CT‐SRGs and CS‐SRGs respectively. Furthermore, using CT‐SRGs to perform CMap searching, we validated the candidate small molecules predicted to regulate SRGs through ex vivo CD34^+^ cell culture and following FACS analysis. We found small molecule cucurbitacin I can efficiently expand HSCs ex vivo while maintaining its stemness. Our results demonstrate SRGs revealed by scRNA‐seq can provide helpful insights for understanding the stemness of HSCs.

## 2 METHODS

### 2.1 Enrichment of CD34^+^ cells from human CB and mPB samples

Human CB and mPB samples were obtained with informed consent from health donor. Mononuclear cells (MNCs) were obtained by centrifugation on Lymphoprep medium, MNC was enriched for CD34^+^ cells selection with the CD34 Microbead kit and LS column using MACS magnet technology (Miltenyi). The sorted CD34^+^ cells were subjected to downstream experiments.

### 2.2 Cell culture and scRNA‐seq

Fresh CD34^+^ cells were immediately cultured ex vivo or used for single‐cell RNA‐seq (scRNA‐seq). For cell culture, CD34^+^ cells were resuspended in SCGM medium (Cellgenix) with the following recombinant hematopoietic cytokines: recombinant human stem cell factor (rhSCF) 100 ng/ml, recombinant human thrombopoietin (rhTPO) 100 ng/ml, recombinant human fms‐related tyrosine kinase‐3 ligand (rhFlt3‐L) 100 ng/ml and cultured in 24‐well tissue culture plates at 37°C in an atmosphere of 5% CO_2_ for 48 h (Thermo Fisher). Then, cells were collected for scRNA‐seq. scRNA‐seq were performed by the DNBelab C4 platform.^26^ In brief, single‐cell suspensions were used for droplet generation, emulsion breakage, beads collection, reverse transcription and cDNA amplification to generate barcoded libraries. Indexed libraries were constructed according to the manufacturer's protocol. The sequencing libraries were quantified by QubitTM ssDNA Assay Kit (Thermo Fisher Scientific; #Q10212). DNA nanoballs (DNBs) were loaded into the patterned nano arrays and sequenced on the ultra‐high‐throughput DIPSEQ T1 sequencer using the following read length: 30 bp for read 1, inclusive of 10 bp cell barcode 1, 10 bp cell barcode 2 and 10 bp unique molecular identifier (UMI), 100 bp of transcript sequence for read 2 and 10 bp for sample index.

### 2.3 Quality control of scRNA‐seq data

The DNBelabC Series HT scRNA analysis Software Suite (v.1.0.0) (https://github.com/MGI‐tech‐bioinformatics/DNBelab_C_Series_HT_scRNA‐analysis‐software/tree/version1.0) was applied to perform sample demultiplexing, barcode processing and single‐cell 3′ UMI counting with default parameters. The read structure is paired‐end: the Read1 has 30 bases, of which the 1^st^–20th bases are cell barcodes, and the 21st–30th bases are UMIs; the Read2 comprises 100‐bp cDNA sequences. The PISA software (https://github.com/shiquan/PISA) was used to parse raw reads into FASTQ+ format based on the library structure and check cell barcodes with the allowed list if the hamming distance is less than or equal to one. Processed reads were then aligned to the UCSC hg38 human genome using splicing‐aware aligner STAR.^77^ with default parameters. Obtained SAM files were transformed into BAM format and annotated with a reference gene set using PISA. The UMIs in reads with the same cell barcode and gene annotation containing 1‐bp mismatch were corrected to the most supported one. Gene‐cell metrics were generated for advanced analysis of valid cells that were automatically recognised according to the UMI number distribution of each cell.

The R (v.3.6.3) package Seurat (v.3.2.1)^49^, ^78^ was used to perform the following steps: (1) quality control of three indicators: the number of genes expressed per cell, the number of UMI and the proportional distribution of mitochondrial RNA to screen high‐quality cells for subsequent analysis.^79^, ^80^ As the number of genes expressed per cell varied greatly, we selected Tukey's test method^81^ to remove cells with abnormal gene numbers. Cells that expressed genes lower than Q1−IQR or higher than Q3+IQR were removed. Meanwhile, cells with a mitochondrial mRNA ratio greater than 10% were also removed; (2) doublets removal. We used an R package ‘DoubletFinder’ (v.2.0.3)^82^ to remove doublets; (3) batch effect removal. We created an integrated data assay of all samples by identifying anchors using ‘FindIntegrationAnchors’ function; (4) data normalisation was performed using ‘NormalizeData’ function with scaling factor 10,000 and then log‐transformed the data. (5) Detection of 4000 highly variable genes (HVGs) by ‘FindVariableFeatures’ function with ‘vst’ method; (6) scaling of the features by ‘ScaleData’ function to get a unit variance and zero mean of all samples.

### 2.4 Dimensionality reduction and cell cluster

The final cell‐gene matrix was introduced into the Seurat (v.3.2.1) package to create a Seurat object followed by employing the ‘CreateSeuratObject’, ‘NormalizeData’, ‘FindVariableFeatures’, ‘ScaleData’ and ‘RunPCA’ functions, to normalisation, scaling and dimensionality reduction successively.^83^ We perform principal component analysis (PCA) on the previously determined variable features by ‘RunPCA’ function with top 40 significant PCs that represent a robust compression of the dataset. Next, we applied a graph‐based clustering approach to construct a shared nearest neighbour graph for a given dataset by ‘FindNeighbors’ function between every cell, and we optimised the modularity function to determine clusters by ‘FindClusters’ function with resolution set to 0.6. Finally, we used UMAP to learn the underlying manifold of the data and place similar cells together in low‐dimensional space.

### 2.5 Cell type annotation

Cell type annotation is divided into two steps: preliminary annotation and manual correction based on existing marker genes.

### 2.6 Preliminary annotation

We executed biologically related pairwise differential gene expression analysis between duos of clusters by ‘FindAllMarkers’ function with ‘min.pct’ equal to 0.2 and ‘logfc.threshold’ set to 0.25, to identify marker genes of each cluster and examine the quantitative changes in the expression levels between the clusters. Then, we compared the marker genes of each cluster with marker genes of defined cell types in the published papers to demonstrate the accuracy of the preceding steps of dimensionality reduction and cell clustering.

### 2.7 Manual correction

Because the marker genes of HSCs and their downstream progeny cells are still uncertain, we additionally collected eight bulk RNA‐seq datasets^33^, ^34^, ^35^, ^36^, ^37^, ^38^, ^43^, ^84^ to improve the cell definition. Detailed procedures are as follows: (1) Using GEO2R, a NCBI online tool, to calculate gene expression levels of reference datasets, respectively; (2) selected top 500 significantly up‐regulated genes as biomarkers of each cell type; (3) performed hypergeometric distribution test between differentially expressed genes (DEGs) of each dataset and current data and assigned the cell type based on the significance of *p* value. The heatmap was created using the R package ‘pheatmap’ with enriched Log_10_(*p* value).

### 2.8 Differential gene expression analysis

We executed biologically related pairwise differential gene expression analysis between duos of samples to identify marker genes and to examine the quantitative changes in the expression levels between the samples in HSCs. We calculated the DEGs by applying the ‘FindMarkers’ function (Wilcoxon rank‐sum with adjusted *p* values for multiple testing with the Benjamini–Hochberg correction). We filtered out the obtained DEGs by setting ‘min.pct’ to 0.2, so that a gene is expressed in at least 20% of the cells in one of the two tested groups. A gene was considered significant with adjusted *p* < .05 and logFC > 0.25.

### 2.9 RNA velocity analysis

By distinguishing between unspliced and spliced mRNAs, RNA velocity, the time derivative of the gene expression state, can be directly estimated. Thus, we utilised velocyto^44^ to compute the rate of transcriptional alteration of each cell. The BAM files of each biological replicate were introduced into dropEst (https://github.com/kharchenkolab/dropEst)^85^ output pipeline to produce 10x‐like BAM files, and were then transformed to standard loom files containing annotated spliced and unspliced reads using velocyto.py (http://velocyto.org/velocyto.py/tutorial/index.html). These files were then merged using python package ‘loompy’ and finally, we employed pagoda2 (https://github.com/kharchenkolab/pagoda2) to obtain cell clusters embedding, and then visualise the RNA velocity by R package ‘velocyto.R’.

### 2.10 Developmental trajectory inference analysis

The Monocle2 (v.2.14.0)^42^, ^86^ algorithm with the core SRGs was applied to order all cells in pseudo time. By creating an object with parameter ‘expressionFamily = negbinomial.size’, regressed out the batch effect using the ‘reduceDimension’ function with the method of DDRTree, with parameter ‘residualModelFormulaStr’ setting to exclude technology influence and with default ‘reduction_method’ to achieve dimensionality reduction. Cell differentiation trajectory was successfully built based on the above steps.

Next, the BEAM function was used to detect genes that separate cells into the considered cell branches. We used the ‘plot_multiple_branches_heatmap’ function to separate the branch‐related gene set with a q‐value less than or equal to 10e^−4^ and the ‘num_clusters’ = 3.

### 2.11 Gene ontology terms enrichment analysis

Gene ontology (GO) enrichment analysis was performed on the given gene set. The enrichGO function of the ‘clusterProfiler’.^87^ R package was used to do enrichment analysis. Terms with the *q* value < 0.05 corrected by FDR were considered statistically significant.

### 2.12 Cell cycle scoring

According to the marker genes directly related to S phase and G2M phase in human cells, we used the ‘CellCycleScoring’ function in Seurat (v.3.2.1) to score the cell cycle phase of each single cell. This function calculated the cell cycle score based on the expression of previously published canonical marker genes.^88^ The single cells highly expressing G2/M‐ or S‐phase markers were scored as G2/M‐ or S‐phase cells, respectively, and those not expressing any of the two categories of genes were scored as G0|G1 phase. We stored S and G2/M scores in Seurat object meta data, along with the predicted classification of each cell in either G2M, S or G1 phase for advanced visualisation.

### 2.13 Prediction of transcription factor regulons

To predict transcription factor regulons, we utilised R package SCENIC (v.1.1.3)^50^, ^89^ to identify potential targets of each TF based on co‐expression to infer gene co‐expression networks with default parameters by ‘GENIE3/GRNBoost’ (https://github.com/aertslab/GRNBoost) and used ‘cisTarget’ database (https://resources.aertslab.org/cistarget/) to analyse transcription factor binding motifs based on DNA‐motif analysis. There are two types of regulons in SCENIC, one is ‘core TF name’_ ‘extended’_ ‘number of target genes’, like ‘HLF_extended_15 g’, representing a gene regulatory network composed of transcription factors and all target genes, the other is ‘core TF name’_ ‘number of target genes’, like MECOM (29 g), on behalf of a gene regulatory network containing transcription factors and high confidence target genes (namely, genes with highConfAnnot = TRUE). ‘AUCell’ (https://github.com/aertslab/AUCell) was applied to generate AUC scores of all cells, which reflects the regulons activity in each cell, to identify cells with active gene sets in scRNA‐seq data. Generated AUC scores of cells were used in downstream visualisation steps.

### 2.14 CMap analysis

Connectivity Map (CMap)^57^ is a database containing expression changes of over 1500 genes in multiple cell types treated with about 5000 small molecule compounds and 3000 genetic reagents. CMap also provide a cloud‐based software platform, CLUE (https://clue.io/query), for the analysis of perturbational datasets generated using gene expression (L1000) and proteomic (P100 and GCP) assays. To understand the relationship between signatures (gene expression profiles) and perturbagens (small molecule), we used Gene Expression (L1000) in Query, a tool of CLUE, with default query parameters as follows: (1) select the ‘Touchstone’, ‘Latest and select’ and ‘Individual query’ parameters; (2) paste the gene into the ‘Up‐Reguleated genes’ dialog box and checked the correct gene name; and (3) submit the query and download the results of ‘DETAILED LIST’ and ‘HEAT MAP’ for advanced screening analysis.

### 2.15 HSCs signature scoring

We collected the top 250 high expressed genes as HSC marker genes of each two published papers^37^, ^38^ as signature. The relative expression of each signature was scored in two SRGs sets and HSC‐LDGs through gene set variation analysis (GSVA; https://www.bioconductor.org/packages/devel/bioc/vignettes/GSVA/inst/doc/GSVA.html)^90^ of each HSC cell, respectively. Violin plots were drawn using the R package ‘ggpurb’ (https://rpkgs.datanovia.com/ggpubr/).

### 2.16 Cells treated with candidate small molecules

Ten thousand CD34^+^ cells from CB and mPB were cultured in SCGM medium (Cellgenix) with the following recombinant hematopoietic cytokines: recombinant human stem cell factor (rhSCF) (100 ng/ml), recombinant human thrombopoietin (rhTPO) (100 ng/ml) and recombinant human fms‐related tyrosine kinase‐3 ligand (rhFlt3‐L) (100 ng/ml). Cells were cultured in 24‐well tissue culture plates at 37°C in an atmosphere of 5% CO_2_ and treated with candidate small molecules NVP‐BEZ235 (10 nM), cucurbitacin I (100 nM), fenretinide (100 nM) and calmidazolium (500 nM). Medium were changed every two days and candidate small molecules were added.

### 2.17 FACS analysis

Sorted CD34^+^ cells without treatment or treated with small molecules at day 6 were collected, washed in DPBS, and then incubated with fluorescence conjugated antibody CD34 (Biolegend) and CD38 (BD) at 4°C for 30 min, washed and resuspended in DPBS for FACS analysis.

## 3 RESULTS

### 3.1 Identification of cell populations in human CD34^+^ cells

**FIGURE 1:**
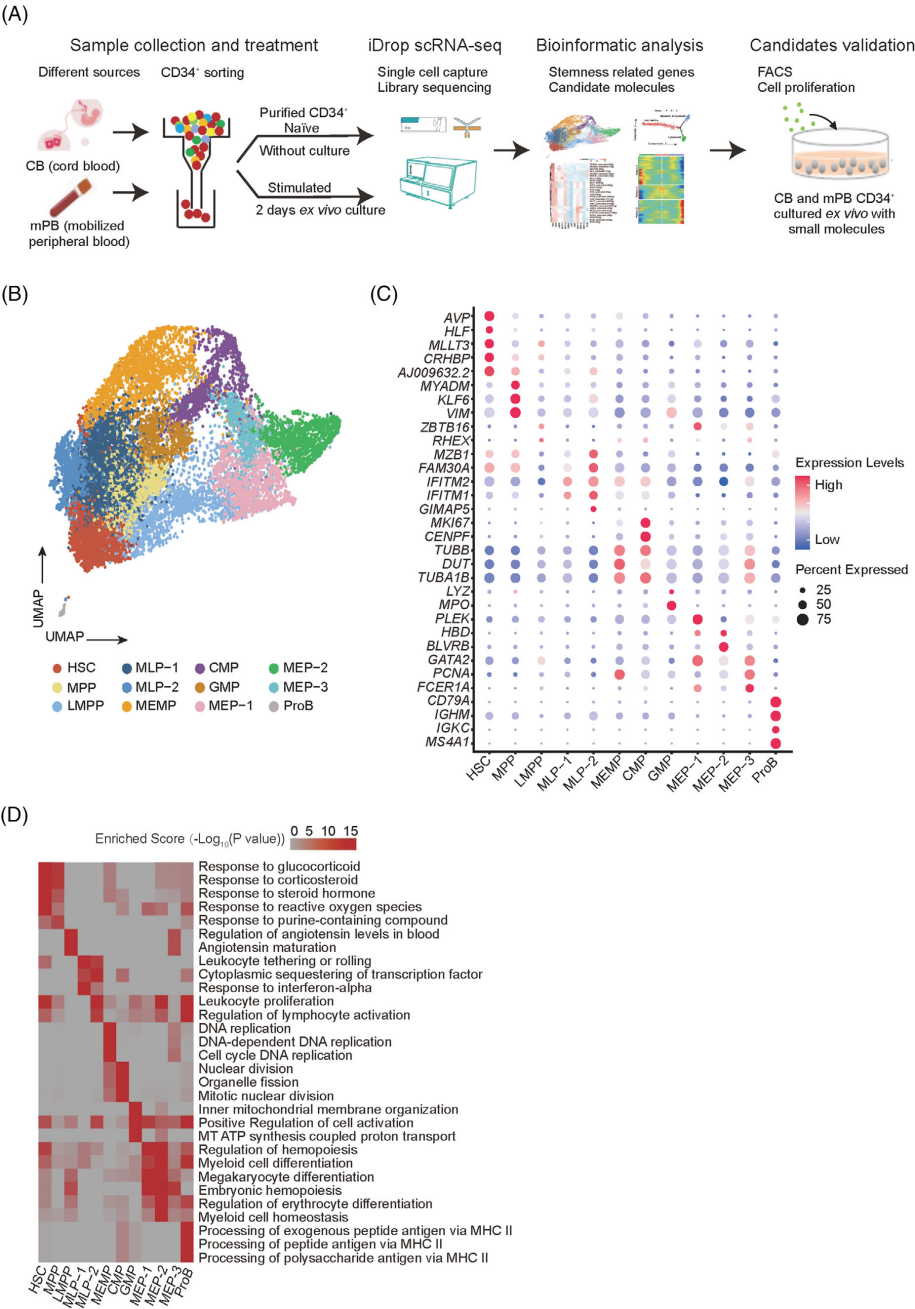
Single‐cell transcriptome landscape of naïve and stimulated human CD34^+^ cells from cord blood (CB) and mobilised peripheral blood (mPB). (A) Schematic diagram of the overall experimental design. (B) All CD34^+^ cells sorted from all eight samples assigned to specific lineages by k‐nearest neighbour (KNN) analysis are illustrated in the same UMAP space generated from the data. (C) Dot plot displaying the expression levels of representative marker genes of each cell type. The marker genes are defined as genes that are significantly up‐regulated in one cell type compared with other cell types. The node size positively correlates with the proportion of a given type of cells expressing a given marker gene. The colour keys from blue to red indicate the relative gene expression level from low to high. (D) Enrichment levels of the top enriched GO terms of marker genes in each cell type. The colour keys from grey to red indicate the −log_10_‐transformed *p* value from low to high

To decipher the differences between CD34^+^ cells from CB and mPB and between the naïve and cultured CD34^+^ cells, we collected CB and mPB CD34^+^ cells from fresh (termed naïve) or cultured for 2 days (termed stimulated) conditions and captured their single‐cell transcriptomes using a massively parallel single‐cell library preparation technique—DNBelab C4.^26^ The overall experimental design is shown in Figure 1A.

We collected two biological replicates for each group, and after quality control we obtained 16 196 cells in total, with an average of ∼3300 genes (∼11 600 UMIs) per cell (Figure S1A). The cell number (replicate1, replicate2) of each group is (4135, 4155) for CB CD34^+^ naive, (1773, 1484) for CB CD34^+^ stimulated, (890, 995) for mPB CD34^+^ naïve and (1272, 1492) for mPB CD34^+^ stimulated. Global correlation analysis revealed a strong correlation between biological replicates, and samples in the same culture conditions had higher transcriptome similarities than samples from the same source (Figure S1B). To get an integrated single‐cell transcriptome map of CD34^+^ cells, we performed graph‐based clustering of the dataset, and found that almost all cells (99.8%, 16 171 of 16 196) were CD34 positive (Figure S1C), which was consistent with our CD34^+^ cell sorting procedure (Figure S1D). Meanwhile, we analyse the overall transcriptional states difference between CB and mPB. The up‐regulated GO terms in CB CD34+ naïve include *aerobic respiration* and *oxidative phosphorylation*, and up‐regulated GO terms in mPB CD34+ naïve are *protein folding in endoplasmic reticulum* and *alternative mRNA splicing via spliceosome* and so on (Figure S1E), suggesting CB CD34+ naïve may have a higher metabolic rate that facilitates the cell proliferation. This is consistent with the fact that CD34^+^ cells from CB have a stronger regeneration capacity. In summary, these results demonstrated that our transcriptome dataset had good quality for the subsequent analysis.

Next, to characterise the human HSC population and its functional signalling pathways, we separated the total 16 196 cells into 12 populations with resolution set to 0.6 (see *Methods*) and collected the top highly expressed marker genes of each population to define cell types (Tables S1 and S2). Based on these marker genes, we were able to assign the populations with distinct cell identities, including HSCs, MPPs, as well as myeloid (MEMPs, CMPs, GMPs), erythroid (MEPs) and lymphoid (LMPPs, MLPs, ProBs) lineages (Figure 1B). The replicates in the same group showed a similar pattern for cell composition (Figure S1F) and the frequency of mRNA expression of these 12 populations were comparable (Figure S1G). Many previously reported marker genes were also confirmed in our data (Figures 1C and S2 and Table S1), such as *AVP*, *MLLT3*, *HLF* and *CRHBP* of HSCs.^4^, ^22^, ^27^, ^28^, ^29^, ^30^ *ZBTB16* of lymphoid‐primed multipotent progenitors (LMPPs),^27^ *TUBB*, *DUT* and *TUBA1B* of megakaryocyte–erythroid–mastcell progenitors (MEMPs).^31^ *MPO* and *LYZ* of granulocyte–monocyte progenitors (GMPs),^22^, ^27^, ^32^ *HBD* and *GATA2* of megakaryocyte–erythroid progenitors (MEPs)^22^, ^27^ *IGKC* and *MS4A1* of B cells progenitors (ProBs).^31^ To further validate the accuracy of cell type identification, we used a hypergeometric distribution test to evaluate the consistency between marker genes of cell clusters in our data and the top 500 up‐regulated genes of cell types in seven published papers.^30^, ^33^, ^34^, ^35^, ^36^, ^37^, ^38^ By this analysis, we also observed a high consistency between these genes, particularly in HSC populations, providing further evidence supporting the cell type identification (Figure S3).

To further confirm the characteristics of these 12 cell types, functional enrichment analysis of the marker genes was performed (Figure 1D). Up‐regulated genes in HSC/MPPs were related to *purine metabolic process* as well as cellular stress response, including *response to glucocorticoid*, *response to corticosteroid* and *response to steroid hormone*. Those have been reported as signature pathways enriched in HSCs in previous studies.^39^, ^40^, ^41^ In contrast, up‐regulated genes in downstream progenitors were enriched for cell differentiation and cell activation related pathways, such as *leukocyte proliferation*, *myeloid cell differentiation* and *megakaryocyte differentiation*, in agreement with the cell development process and cell‐cycle progression (Figure 1D and Table S3). Taken together, we obtained high‐quality scRNA‐seq data from 16 196 sorted CD34^+^ cells from naïve and stimulated CB and mPB and identified 12 cell types including HSCs. Our data are consistent with previous reports, while providing a more comprehensive reference map for investigating the underlying mechanism of human HSCs.

### 3.2 Differentiation trajectory of human HSCs

**FIGURE 2:**
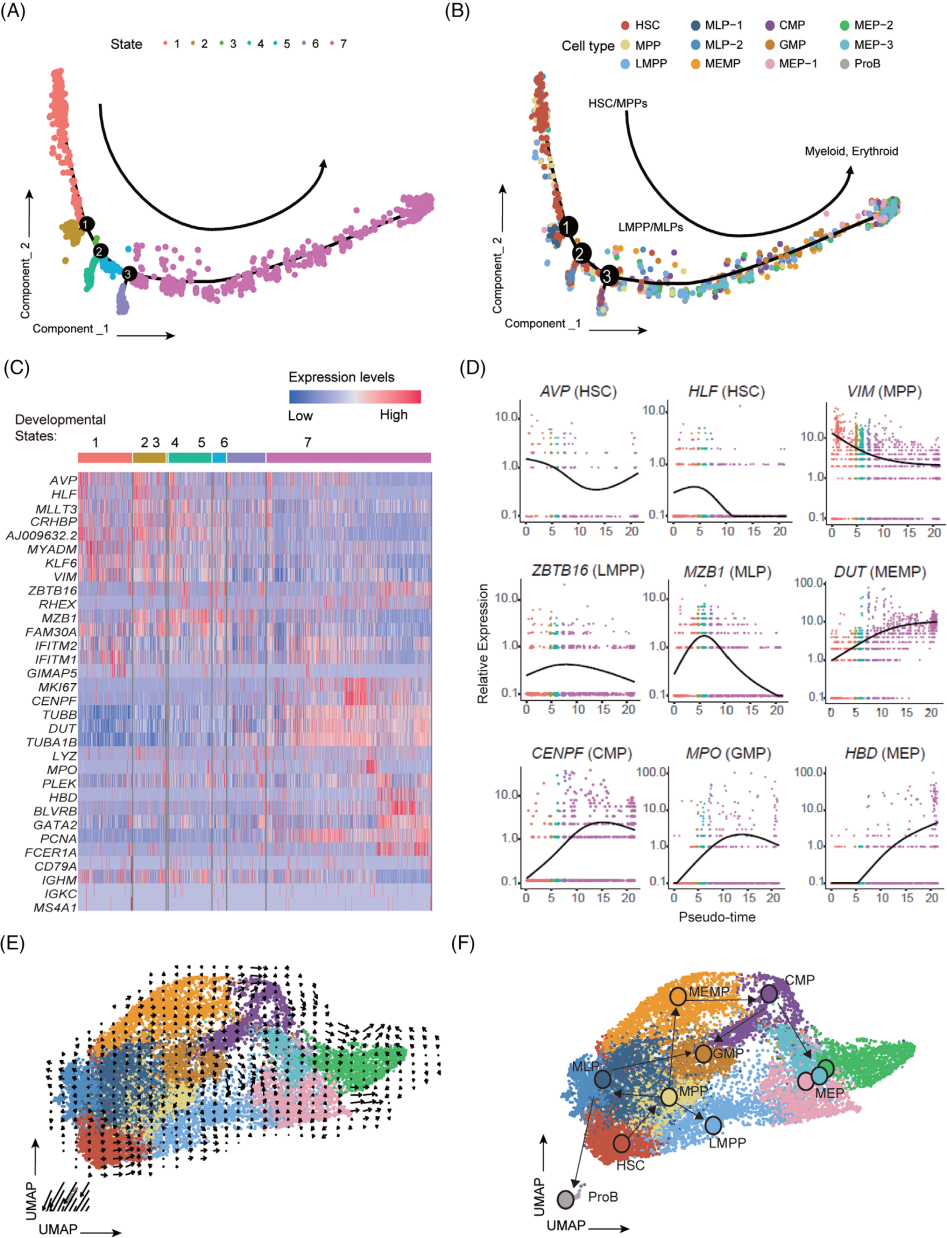
Developmental trajectory analysis of human CD34^+^ cells. (A) Developmental trajectory of all 16 196 CD34^+^ cells reveals seven states recognised by Monocle2. Each dot represents one cell and different colours represent different states. Cells from the same state have similar pseudo time along the trajectory, while the arrow indicates the direction of the trajectory. The branch points of the trajectory are marked by black circles with numbers. (B) Cell type assignment along the developmental trajectory. Each dot represents one cell and different colours represent different classes of progenitor cells. HSC and MPP are mainly located in State1, LMPP and MLPs lie at State2‐4, and myeloid and erythroid progenitor cells (MEMP, CMP, GMP and MEPs) are gathered in State5‐7. (C) Heatmap showing the expression of marker genes (shown in Figure 1C) in seven developmental states, whereas the colour keys from blue to red indicate the relative gene expression levels from low to high. (D) Expression levels of representative marker genes along developmental pseudo time. (E and F) Amalgamated (E) and fitted (F) lineage trees showing the developmental routine from HSCs to multipotent progenitors and downstream lymphoid, myeloid and erythroid lineages. Arrows represent developmental directions, and each circle in (F) represents a cluster.

To further validate the accuracy of our reference map, we used Monocle^42^ to conjecture the differentiation trajectory with total 16 196 cells and checked whether our 12 cell types exhibit similar patterns with previous studies, which found that HSCs first differentiated into MPPs, and then into LMPPs and other progenitor cells.^2^, ^43^ In our results, seven state cells were distributed along the trajectory and we found most HSCs/MPPs were located near the tips of the trajectory, while other cells were distributed amongst the six branches (Figures 2A and B and S4A), in agreement with previous reports. Consistently, PCA revealed that the cell types defined as adjacent developmental states in our map were clustered together, such as HSCs, MPPs and LMPPs which were all upstream progenitors (Figure S4B). To determine the lineage affiliation of these branches, we checked the expression patterns of some marker genes. As we expected, the expression levels of *AVP, HLF* and *VIM*, all related to self‐renewal potential and quiescence in HSCs and MPPs^22^, ^28^, ^30^ were decreased over the pseudo time. *ZBTB16* and *MZB1*, the marker genes of LMPPs and MLPs, were up‐regulated at the early stage but then decreased later along the pseudo‐time, in agreement with the position of LMPPs and MLPs on the trajectory. Myeloid and erythroid lineages such as MEMPs, CMPs, GMPs and MEPs were mainly situated at the end of the trajectory (States5–7) with high expression of their lineage marker genes such as *DUT, CENPF, MPO* and *HBD* (Figures 2C and D).

To further confirm the differentiation trajectory inferred, we used RNA velocity^44^ to project all cells into a two‐dimensional UMAP space with arrows showing the direction and the speed of differentiation (Figures 2E and S4C). When a developmental routine was finally fitted (Figure 2F), we found that it was consistent with the current classical model of lineage determination in human hematopoietic hierarchy^45^, ^46^ thus further illustrating the accuracy of cell annotation and differentiation trajectory of our data.

### 3.3 The characteristics and gene regulatory networks of human HSCs

**FIGURE 3:**
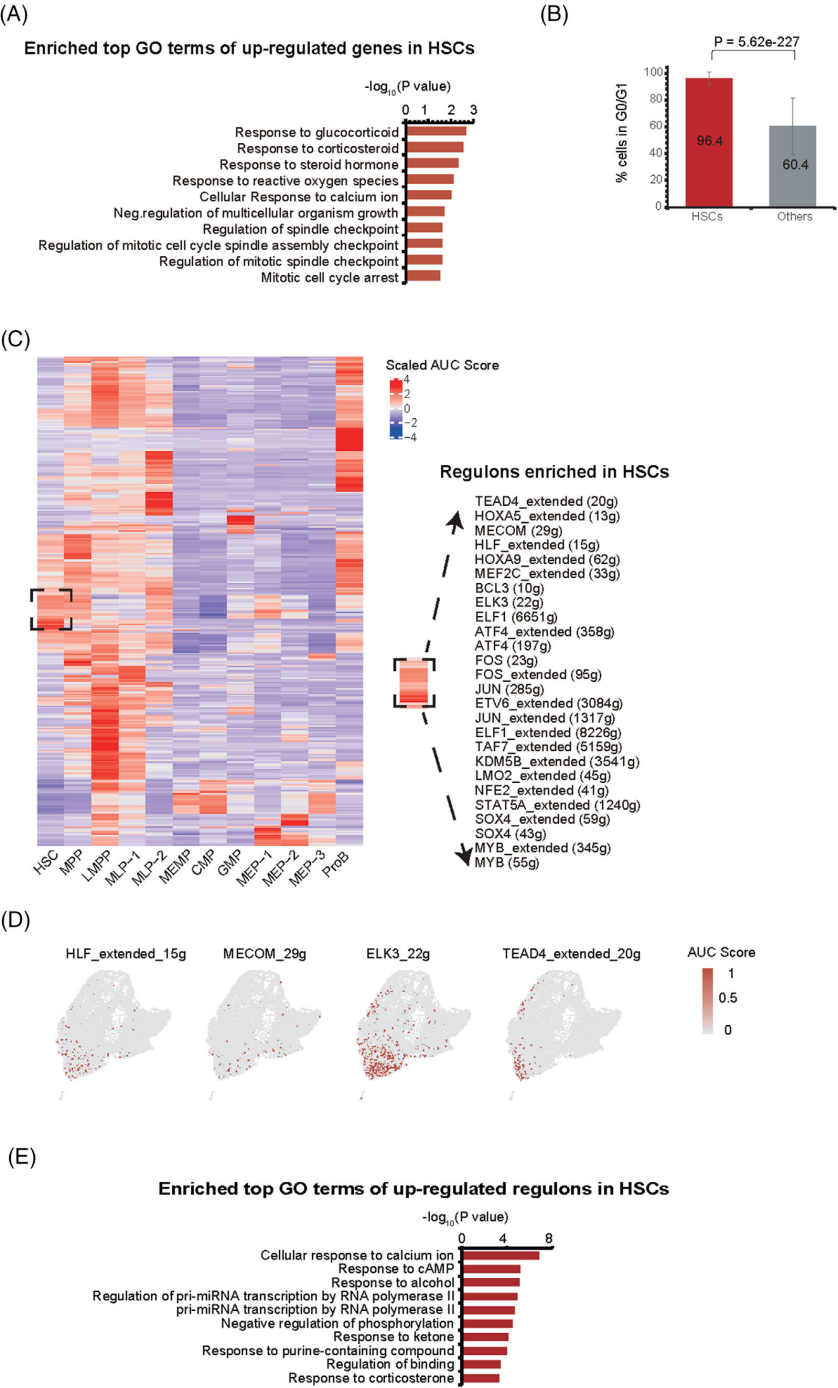
Cell cycle and gene regulatory networks of human HSCs. (A) Enriched GO terms of up‐regulated genes in HSCs compared with other cell types. (B) The percentage of HSCs and other cells in G0/G1 stage. *p* Value was calculated by Fisher's exact test. (C) Heatmap showing up‐regulated regulons in each cell type compared with other cell types (left). Up‐regulated regulons in HSCs are listed in right. The colour keys from blue to red indicate scaled area under curve (AUC) value from low to high. The higher scaled AUC value a regulon has, the more enriched regulon. (D) UMAP plot showing the representative regulons of HSCs. Cells are coloured by AUC value of each regulon. The colour keys from grey to red indicate AUC scores from low to high. (E) Enriched GO terms of up‐regulated regulons in HSCs. In this figure, the HSCs indicates the HSC cells from all collected samples regardless of cell source or culture time.

To further characterise the human HSCs in our data, we did GO term analysis of the up‐regulated genes of HSCs compared with other cell types. We found pathways associated with cell cycle as well as mitosis, such as *regulation of spindle checkpoint*, *regulation of cell cycle spindle assembly checkpoint* and *mitotic cell cycle arrest*, were present as the most significant ones, which also includes other GO terms such as *response to glucocorticoid* and *response to corticosteroid* (Figure 3A). The cell cycle activity of CD34^+^ cells over the lifetime is dynamic, and it reflects the requirements of the organism at different developmental points.^47^, ^48^ Thus, we calculated cell cycle phase scores based on canonical markers by Seurat^49^ and found that HSCs were significantly enriched in G0 and G1 phases (96.4%, *p* value = 5.62E−227; Figure 3B and Table S4) when compared with other cell types, indicating that most HSCs were in a resting state, in consistent with GO term analysis and previous studies.

Subsequently, we wondered whether some gene regulatory networks (regulons), a collection of genes regulated by common transcript factors (TFs), specifically existed in HSCs. To achieve this, we applied SCENIC^50^ to each cell of the 12 identified cell types. SCENIC recognised 371 activated regulons whose activities were dynamically changed among different cell types (Figures 3C and S5). Interestingly, expression of TFs in several identified regulons, like *HLF*, *MECOM* and *ELK3*, were also enriched in HSCs and MPPs (Figures 3C and D and Table S2). In addition, the functions of these TFs and their target gene sets were significantly enriched in *response to corticosterone* and *response to purine‐containing compound* (Figure 3E), which were consistent with those of HSC marker genes (Figures 3A and 1D). Thus, using regulons analysis, we confirmed previous reported TFs, such as HLF and MECOM,^51^ and their target genes, were also presented in HSCs of our data.^22^, ^28^ Besides, we also found many novel regulons worthy of further investigations, including *FOS*, *MEF2C*, *TEAD4*, *ELK3* and *HOXA9* (Figures 3C and D). Thus, our single‐cell data not only captured the previous HSC signatures, but may also provide new hints for investigating HSCs in general.

### 3.4 Identification of SRGs probably controlling stemness of human HSCs

**FIGURE 4:**
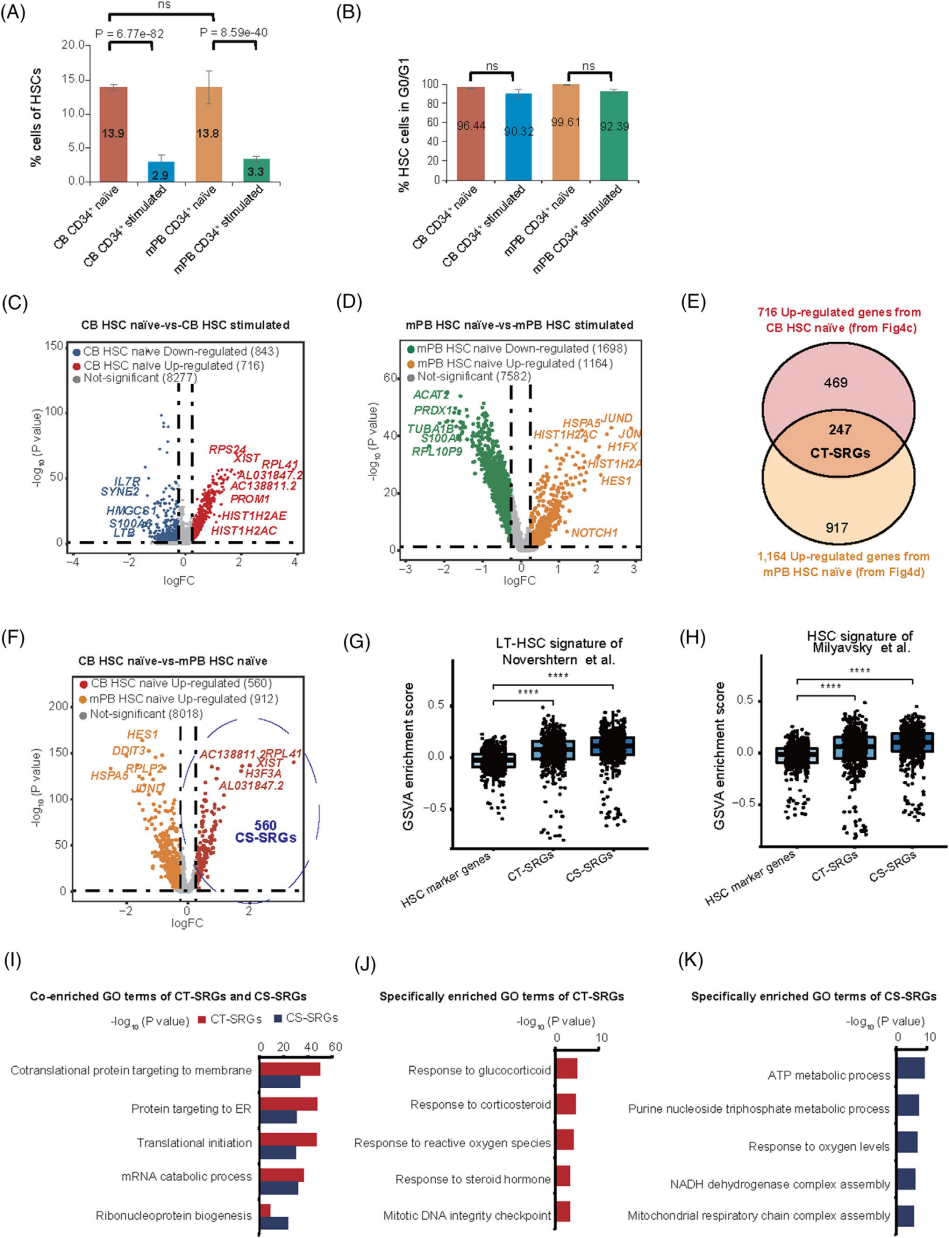
Identification and characterisation of stemness‐related genes (SRGs) related to culture time and cell source. (A) Bar graphs displaying the proportion of HSCs in naïve and stimulated CD34^+^ cells from CB and mPB (*n* = 2). *p* Value was calculated by Fisher's exact test. ns means not significant. (B) Bar graphs displaying the percentage of HSC cells in G0/G1 stage per sample (*n* = 2). *p* Value was calculated by Fisher's exact test, and there was no significant difference among these groups. (C) Volcano plots showing gene expression changes of HSCs between naïve CB and stimulated CB. The CB HSC naïve and CB HSC stimulated indicates the HSC cells from naïve and stimulated CB source respectively. (D) Volcano plots showing gene expression changes of HSCs between naïve mPB and stimulated mPB. The mPB HSC naïve and mPB HSC stimulated indicates the HSC cells from naïve and stimulated mPB source respectively. (E) Venn diagram showing 247 CT‐SRGs obtained from the intersection of 716 up‐regulated genes in CB HSC naïve (C) and 1164 up‐regulated genes in mPB HSC naïve (D). (F) Volcano plots showing gene expression changes of HSCs between naïve CB and naïve mPB. The HSCs naïve indicates the HSC cells from CB and mPB without cell culture (naïve). Cell Source‐related SRGs (CS‐SRGs, *n* = 560), marked by the purple dotted circle, was the up‐regulated genes in CB HSC naïve compared with mPB HSC naïve. (G and H) Violin plots showing the GSVA enrichment scores of intersection genes between “HSC marker genes,” “CT‐SRGs,” “CS‐SRGs” and signature genes of LT‐HSC or HSC reported by Novershtern et al. (G) and Milyavsky et al. (H). HSC marker genes were the marker genes up‐regulated in HSCs compared with other cell types (shown in Table S2), whereas CT‐SRGs and CS‐SRGs were described in (E) and (F). The horizontal axis represents different gene sets, and the vertical axis represents GSVA enrichment scores. *p* Value was calculated by two‐sided Wilcoxon rank‐sum test. (I) Common enriched GO terms of CT‐SRGs and CR‐SRGs. GO terms enriched in CT‐SRGs are shown in red, whereas GO terms enriched in CS‐SRGs are shown in blue, the coloured bars indicate −log_10_‐transformed *p* values. (J) Specifically enriched GO terms of CT‐SRGs compared with CS‐SRGs. (K) Specifically enriched GO terms of CS‐SRGs compared with CT‐SRGs.

The purpose of this study is to understand the differences of CD34^+^ cells from CB and mPB and before and after culture, and to identify the SRGs. We found that HSCs accounted for 9.9% of all cells profiled in our data, and their composition was significantly decreased under stimulated condition in both CB and mPB samples (Figure 4A and Table S5), in agreement with the reduced stemness of these samples. Interestingly, we noticed that cell sources and 2 days ex vivo culture only slightly affect the cell cycle phase scores (Figure 4B), suggesting quiescent maintenance is still a robust characteristic for all HSCs, even after a short time *ex vivo* culture. To reveal SRGs responsible for stemness maintenance of HSCs, we investigated the transcriptome changes between naïve and stimulated HSCs first. By differential expression analysis, we obtained 247 CT‐SRGs (culture time‐related SRGs), from the intersection of 716 up‐regulated genes in HSCs of CB CD34^+^ naïve and 1164 up‐regulated genes in HSCs of mPB CD34^+^ naïve (*p* value < .05 and ln‐transformed fold change > 0.25) (Figures 4C–E and Tables S6–8). In addition, as previous studies found that CD34^+^ cells derived from CB exhibited a higher level of stemness than mPB,^52^, ^53^, ^54^ we also obtained 560 CS‐SRGs (cell source‐related SRGs) by comparing HSCs of naïve CB to that of naïve mPB (*p* value < .05 and ln‐transformed fold change > 0.25) (Figure 4F and Table S9). GSVA using HSC data sets from two published papers^37^, ^38^ revealed that the overall expression levels of both CT‐SRGs and CS‐SRGs were significantly higher than HSC marker genes (Figures 4G and H), further demonstrating that both CT‐SRGs and CS‐SRGs could better reflect the stemness of HSCs.

Then we asked the commonalities and differences between CT‐SRGs and CS‐SRGs. Interestingly, a large fraction of the intersection genes in CT‐SRGs and CS‐SRGs are enriched in GO terms related to *protein targeting to membrane* (*p* value of CT‐SRGs = 1.94E−50, *p* value of CR‐SRGs = 2.25E−34) and *protein targeting to ER* (*p* value of CT‐SRGs = 1.48E−48, *p* value of CR‐SRGs = 3.26E−31) (Figure 4I), which are signatures for protein synthesis process and translation of ribosomal coding genes (Table S10). It is worth noting that nine genes specifically present in CT‐SRGs, including *AREG*, *CFLAR*, *DDIT4*, *DUSP1*, *FLT1*, *FOS*, *FOSB*, *FOXO3* and *ZFP36*, were significantly enriched in GO terms of *response to glucocorticoid* (*p* value = 8.50E−06) and *response to corticosteroid* (*p* value = 1.96E−05) (Figure 4J), which has been reported to affect HSCs homing and engraftment.^39^ By contrast, we found *purine nucleoside triphosphate metabolic process* (*p* value = 4.90E−12) and *ATP metabolic process* (*p* value = 4.48E−10), which is crucial for HSCs homeostasis in the stress settings,^55^, ^56^ were specifically enriched in CS‐SRGs (Figure 4K).

Taken together, these results demonstrated that CT‐SRGs and CS‐SRGs share common pathways, and both CT‐SRGs and CS‐SRGs exhibit a better consistency with HSC characteristics than unsorted HSC marker genes, indicating their potential function and applications in human HSCs. Furthermore, CT‐SRGs and CS‐SRGs still have their own specifically enriched genes and signalling pathways, suggesting the stemness differences because of cell sources and culture time share distinct mechanism as well.

### 3.5 The differentiation trajectory of human CD34^+^ cells reconstructed using CT‐SRGs

**FIGURE 5:**
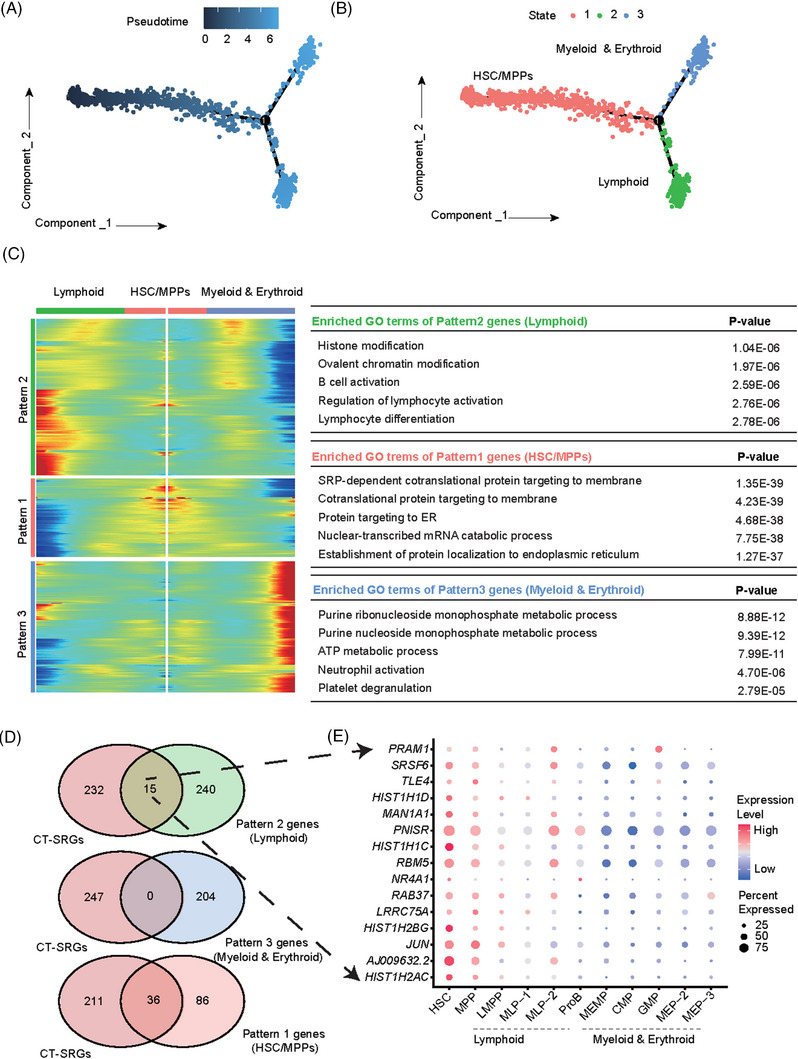
Differentiation and development trajectory of CD34^+^ cells based on CT‐SRGs. (A) The trajectory tree reconstructed using CT‐SRGs (culture time‐related SRGs, *n* = 247). Each dot represents a cell and the colour keys from dark to light indicate the differentiation time from early to late. The branch point of the trajectory was marked with black circle and the number 1. (B) The State branch recognised by Monocle2. Each dot represents a cell and different colour represents different states identified by Monocle2, whereas cells from the same state have similar pseudo time along the trajectory. The branch point of the trajectory was marked with black circle and the number 1. The general localisation of HSC/MPPs, lymphoid (mainly corresponsive to LMPP, MLP‐1, MLP‐2 and ProB) and myeloid and erythroid (mainly corresponsive to MEMP, CMP, GMP, MEP‐1, MEP‐2 and MEP‐3) were named according to the mapping relationship of different cell types in states (Figure S6a) and the expression change of lineage marker gene along the pseudo time (Figure S6b). (C) Heatmap of the key genes involved in branch determination and their functions. The three dynamic expression patterns of genes highly expressed in lymphoid lineage (Pattern 2), HSC/MPPs (Pattern 1) and myeloid and erythroid lineages (Pattern 3) were shown on the left, with specifically enriched GO terms shown on the right. (D) Venn diagram showing shared genes between CT‐SRGs and the genes of three patterns shown in (C). Fifteen genes were shared in CT‐SRGs and Pattern 2 genes (Lymphoid). (E) Dot plot displaying the expression of 15 shared genes in different cell types. The node size represents the cell proportion that expresses the given gene. The colour keys from blue to red indicate the relative gene expression levels from low to high.

During HSCT and HSC‐GT, the ability of CD34^+^ cells to rebuild the hematopoiesis is negatively correlated with the *ex vivo* culture time, therefore to understand the transcriptomic changes during this process, we focused on CT‐SRGs in further analysis. First, we reconstructed the differentiation trajectory of human CD34^+^ cells using CT‐SRGs, and we were amazed to find that State2 and State3 were clearly separated in the developmental trajectory after State1 (Figure 5A). Consistent with the trajectory conjectured based on marker genes (Figure 2B), the majority of HSC and MPP were still located at the top of the trajectory (State1). In contrast, lymphoid progenitors (LMPP, MLP‐1, MLP‐2 and ProB) and myeloid/erythroid progenitors (MEMP, CMP, GMP, MEP‐1, MEP‐2 and MEP‐3) in Figure 1B are mainly located at State2 and State3 respectively (Figure S6a). Thus, we named State1, State2 and State3 as ‘HSC/MPPs’, ‘Lymphoid’ and ‘Myeloid and Erythroid’ (Figure 5B). We further checked the expression changes of marker genes along pseudo‐time. *AVP*, *HLF*, *VIM* and *KLF6* of HSC and MPP were highly expressed in State1, then decreased with the pseudo‐time progression. Oppositely, *MKI67* and *HBD* gradually increased with pseudo‐time and reached the peak in State3, signifying Myeloid and Erythroid lineages may enrich in State3. State2 should be Lymphoid lineage with high expression of *IGKC* and *MS4A1* (Figure S6B). Next, we identified DEGs between the branches to further corroborate the previous results, and we got three gene clusters with different expression patterns across the three states. GO Term analysis of the three gene clusters indicated related pathways were enriched in corresponding states, such as *lymphoid differentiation* in Lymphoid lineage (State2), *cotranslational protein targeting to membrane* in HSC/MPPs (State1) and *neutrophil activation* in myeloid and erythroid lineages (State3) (Figure 5C). A total of 15 CT‐SRGs were up‐regulated in State2, whereas no CT‐SRG was up‐regulated in State3 (Figure 5D). These results indicated that Lymphoid lineage may be closer to HSC/MPPs when compared with Myeloid and Erythroid lineages. In agreement, the 15 CT‐SRGs were up‐regulated in lymphoid progenitors as well as in MPPs and HSCs (Figure 5E). In conclusion, CT‐SRGs may be better reference genes for development trajectory construction. The updated trajectory revealed a closer relationship between HSCs and lymphoid lineage, and could be used to explore new strategies for the maintenance of HSC stemness.

### 3.6 Small molecules modulating CT‐SRG promote cell proliferation and stemness maintenance of human HSCs ex vivo

**FIGURE 6:**
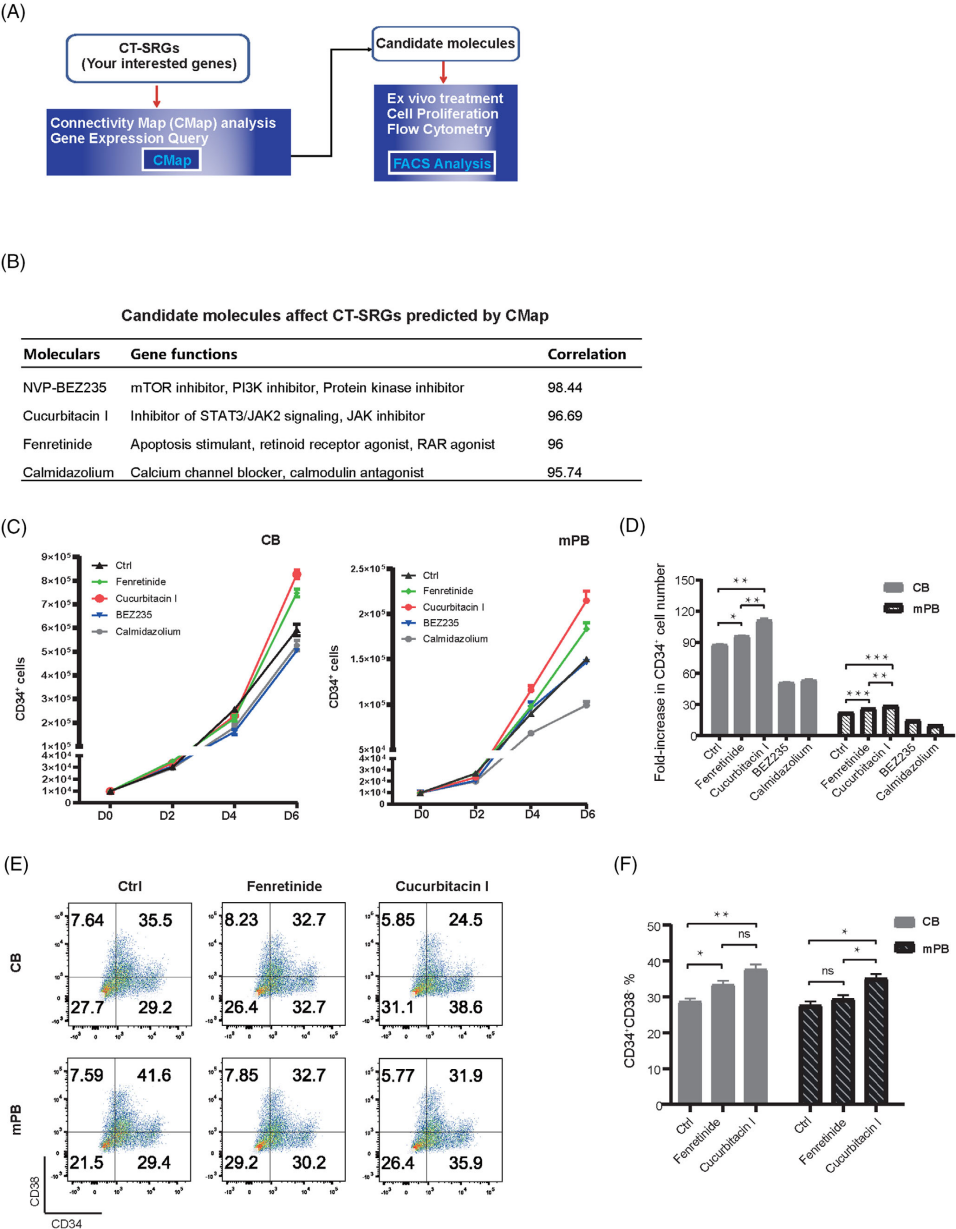
Cucurbitacin I could enhance HSCs proliferation and stemness. (A) The diagram of small molecules screening pipeline. Small molecules that might perturb CT‐SRGs expression were predicted using CMap, and then verified by cell culture and FACS ex vivo. (B) Candidate molecules affecting CT‐SRGs predicted by CMap. The first column is the names of small molecules, the second column is the biological function of corresponding genes, and the third column is the correlation score calculated by CMap. (C) Proliferation of CD34^+^ cells from CB and mPB after treatment with candidate small molecules were calculated at day 0, day 2, day 4 and day 6 after ex vivo culture. Both CB and mPB were cultured with 10 000 initial cells. (D) Fold‐increase in CD34^+^ cell number after 6 days ex vivo culture as compared with input numbers. (E) Flow cytometry plots show CD34^+^CD38^–^ proportions from CB and mPB after 6 days ex vivo culture. (F) Statistic analysis of CD34^+^CD38^−^ proportions after different small molecule treatments. *p* Value was calculated by *t*‐test, *p < .05; **p < .01; ***p < .001; ns, not significant. Error bars indicate standard deviations of triplicate cultures. For fenretinide and cucurbitacin I treatment, 100 nM dose was used.

To check whether CT‐SRGs are important for human HSCs, we used CMap, an online tool kit based on a perturbation‐driven gene expression dataset^57^ to search for candidate small molecules that could affect the global expression levels of CT‐SRGs. The identified small molecules will be tested in the following experiments to see if they can affect the stemness of HSCs (Figure 6A). In sum, we identified 145 candidates of CT‐SRGs. Among them, small molecules function as protein synthesis inhibitors (such as emetine and cephaeline) and glucocorticoid/corticosteroid receptor agonist (dexamethasone) were predicted to positively regulate the expression levels of CT‐SRGs (Table S11). These data are consistent with the above results, that the functions of CT‐SRGs were enriched in protein synthesis process and glucocorticoid/corticosteroid responses (Figures 4I and J). In addition, small molecules that target ATPase, mTOR and MAP kinase pathways, were also characterised as candidates in our screening (Table S11). Importantly, fenretinide, a retinoid receptor agonist, is identified in our CMap screening against CT‐SRGs. And it has been reported to enhance human HSC self‐renewal by modulating sphingolipid metabolism ex vivo.^58^ In summary, small molecules predicted to modulate CT‐SRGs can be candidates capable of regulating human HSC stemness.

To identify other regulators of HSC stemness, we selected three small molecules, NVP‐BEZ235, which is related to the signal of mTOR/PI3K,^59^ cucurbitacin I, which is related to the signal of STAT3/JAK2,^60^ and calmidazolium, which is related to the signal of calcium channel blocker, calmodulin antagonist^61^ (Figure 6B). We treated CB and mPB CD34^+^ cells *ex vivo* with those small molecule candidates, cultured them for an extended time period (up to 6 days) and then measured the CD34^+^ cell proliferation and HSCs percentage. Fenretinide was used as a positive control. When compared with fenretinide treatment and untreated control, only cucurbitacin I, but not other tested molecules, increased the CD34^+^ cell numbers in both CB and mPB after 6 days’ ex vivo culture (Figures 6C and D). More important, when examining a more restricted surface markers of HSCs using FACS, the cucurbitacin I treated cells also exhibited the highest proportion of CD34^+^CD38^−^ cells. These results indicate that cucurbitacin I not only enhances the expansion but may also maintains the stemness of human HSCs from both CB and mPB sources (Figures 6E and F). Taken together, we provided a preliminary application of CT‐SRGs to identify HSCs modulators, and validated that a small molecule cucurbitacin I could enhance human HSC proliferation while maintaining their stemness ex vivo.

## 4 DISCUSSION

CD34^+^ cells from CB and mPB possess distinct ability to reconstruct hematopoiesis after allogeneic stem cell transplantation and HSCs loss stemness during ex vivo culture. Uncovering the underlying mechanism of these phenomena may provide helpful insights to understand HSCs function and to expand HSCs ex vivo without losing stemness. Many previous studies have demonstrated that, scRNA‐seq without cell sorting can capture detailed molecular profiles of all cell populations at single cell level and be used to characterise novel cell clusters and their corresponding signature genes.^22^, ^62^ Using this method, novel cell types have been identified and functionally validated during hematopoiesis.^30^, ^63^, ^64^

In our present study, we collected over 16 000 single‐cell data of CD34^+^ cells from native and stimulated CB and mPB and subsequently performed the bioinformatic analyses and functional assays (Figure 1A). It is worth mentioning that our data shows good quality with a higher average gene number per cell and a lower mitochondrial percentage (Figure S1). We first constructed a reference map and identified 12 populations enriched in specific genes and signalling pathways, including HSCs (Figures 1B–D). Importantly, our data showed consistency with previous studies,^2^ as all classical cell types reported before have their corresponding parts in our data (Figure S3). Furthermore, our cell types exhibit similar patterns of differentiation trajectory with previous studies (Figure 2), illustrating the accuracy of cell annotation and our reference map. Based on these molecular data, we characterised the gene regulatory networks of human HSCs on top of our trajectory map, and found that most HSCs stay in G0 and G1 phase, indicating that cells were in a quiescent state. In agreement, transcription factors critical for the maintenance of HSCs, such as *AVP*, *MLLT3*, *HLF*, *MECOM*, *CD52*, are highly expressed in HSCs. And those TFs and their target gene sets were significantly enriched in *response to corticosterone* and *response to purine‐containing compound* (Figure 3). Besides, novel transcription factors and regulons, not reported to be involved in HSCs, have been revealed, such as *FOS, MEF2C, TEAD4, ELK3* and *HOXA9*. Those factors are candidates for further investigation.

After confirming the validity of our data, we next identified SRGs associated with culture time (CT‐SRGs) and cell source (CS‐SRGs) respectively, and analysed their enriched pathways and potential application for stemness score and trajectory construction (Figures 4 and 5). CT‐SRGs and CS‐SRGs share common signalling pathways involved in *mRNA catabolic process*, *translational initiation* and *ribonucleoprotein complex biogenesis*, suggesting dynamic protein translation and processing may be a common requirement for stemness maintenance. In agreement, regulation of protein synthesis and ribosome biogenesis are strongly coupled to stem cell behaviour, and the translation regulatory mechanisms that affect stem cell function include mTOR signalling, ribosome levels, and mRNA and tRNA features and mounts.^65^, ^66^ Previous studies have demonstrated that protein synthesis is also delicately regulated in HSCs, and disruption of ribosome function or tRNA editing, perturb the HSCs proliferation, differentiation and engraftment.^67^, ^68^, ^69^, ^70^ Here, with scRNA‐seq and a systematic comparison, our results suggest that changes of protein biosynthesis may be also responsible for the stemness decrease of CD34^+^ cell during normal ex vivo culture, and be the reason why CD34^+^ cell from mPB has weaker repopulating capability in transplantation. It will be interesting to see whether modulating these pathways discovered in our study can enhance the HSCs function, although it could be challenging as either reduced or increased protein synthesis impairs HSCs function.^71^ Furthermore, we found CT‐SRGs is specifically enriched in signalling pathways of *response to glucocorticoid* and *response to corticosteroid*, and CS‐SRGs is enriched in *purine nucleoside triphosphate metabolic process* and *ATP metabolic process* by contrast (Figures 4J and K). Therefore, our results suggest that stronger stemness of naïve HSCs is probably due to their higher sensitivity to glucocorticoids. Previous studies already show glucocorticoids act as an activator of *CXCR4* and pre‐treatment of human HSCs with glucocorticoids promote their homing and long‐term engraftment after transplanted into NSG mice.^39^ Thus, naïve HSCs may be also more likely to migrate to the niche through glucocorticoid‐activated CXCR4/SDF‐1 chemotaxis after transplantation. To maintain the long‐term engraftment capability of HSCs during ex vivo culture, pre‐treatment of HSCs with glucocorticoids or overexpressing CXCR4 in HSCs could be possible solutions. Instead, to enhance the stemness of HSCs from mPB, modulating metabolism, particularly the purine metabolites, may be the key points, as HSCs from CB show a stronger purine metabolic process in our data. Consistently, purine metabolites have been reported to play a crucial role for HSC maintenance and their response to stress.^55^, ^56^ It is worth noting that, though SRGs are obtained by comparing cells with stronger stemness to those with weaker stemness, it doesn't mean SRGs are only related to ‘stemness’ or ‘regeneration’, and SRGs may be also related to the primed potential or developmental trajectory of the cells with stronger stemness. Interestingly, we found CT‐SRGs may be better reference genes for construction of the development trajectory of CD34^+^ cells. In this updated trajectory, Lymphoid and Myeloid and Erythroid lineages are clearly separated after HSC/MPPs. Moreover, parts of CT‐SRGs are up‐regulated in both Lymphoid and HSC/MPPs, suggesting Lymphoid lineage have a closer relationship with HSC/MPPs in human (Figure 5). In agreement, a previous study indicates hypoxia can favour production of human lymphoid cells as well as HSCs.^72^ Another study show that lymphoid‐biased progenitors are capable of long‐term survival and can be maintained independently from HSCs after autologous transplantation.^73^ Therefore, it could be interesting to check whether the up‐regulated CT‐SRGs play a role in those lymphoid‐biased progenitors. Meanwhile, as CT‐SRGs are related to culture time, it may be worthy to investigate whether ex vivo culture process affects lymphoid development, which is important for rebuilding adaptive immune system after transplantation.

It is well known that cell–cell interaction is important for niche remodelling and HSC homeostasis.^74^, ^75^ Thus, one pitfall of this study is that we cannot exclude the roles of other cell types in CB and mPB CD34^+^ cells during HSCT. Nevertheless, we selected small molecules modulating CT‐SRGs via a virtual screening process and performed cell proliferation and FACS assays. A small molecule cucurbitacin‐I, from 3 tested candidates, can promote HSCs proliferation while maintaining their stemness (Figure 6). Cucurbitacin I is a regulator of multiple signalling pathways and is used in cancer treatment.^76^ However, its function in human HSCs has not been clarified. Here, our data suggest cucurbitacin I may be a novel therapeutic molecule to expand HSCs ex vivo, though its role on HSCs require further validation in mice transplantation experiment. Our findings suggest that CT‐SRGs (247 genes), as well as CS‐SRGs (560 genes), could be used as a database to select small molecules that modulate human HSCs function. The selected candidates are worthy for subsequent functional validations.

Overall, our studies indicate ex vivo culture may more likely to affect the homing and engraftment capability of HSCs, while HSCs from CB share different metabolic regulations from that of mPB. SRGs revealed by our scRNA‐seq can be a valuable database to identify new candidates for functional HSC expansion. With further investigation, our results may guide researchers and clinicians to further optimise the HSCs processing in both gene therapy and clinical applications.

## CONFLICT OF INTEREST

All the authors declare no competing interests.

## FUNDING INFORMATION

This research are supported by grants from National Natural Science Foundation of China (NSFC) (31970816), Shenzhen Key Medical Discipline Construction Fund (SZXK034) and Shenzhen Fund for Guangdong Provincial High‐level Clinical Key Specialties (SZGSP012).

## Supporting information

Supporting Information

Supporting Information

Supporting Information

Supporting Information

Supporting Information

Supporting Information

Supporting Information

## Data Availability

The scRNA‐seq data generated in this study have been deposited in the CNSA (https://db.cngb.org/cnsa/) of CNGBdb with accession code CNP0000978.
