# Supplementary figures and images for: Stemness‐related genes revealed by single‐cell profiling of naïve and stimulated human CD34+ cells from CB and mPB
Source: Clin Transl Med. 2023 Jan 22;13(1):e1175. doi: 10.1002/ctm2.1175 (PMC9868212; doi:10.1002/ctm2.1175)

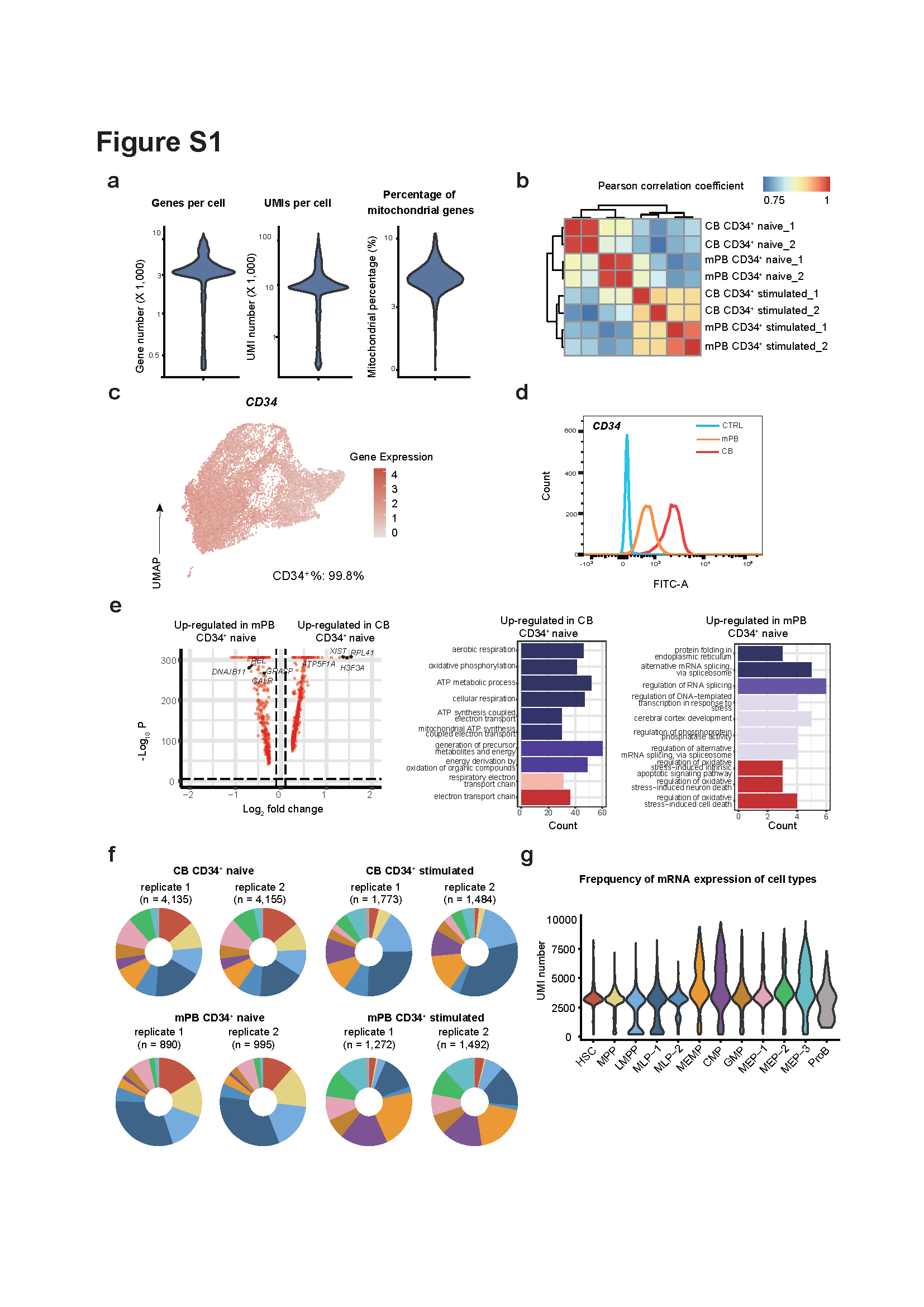

Supplement: Supplementary file 2 — Supporting Information [file CTM2-13-e1175-s004.png]

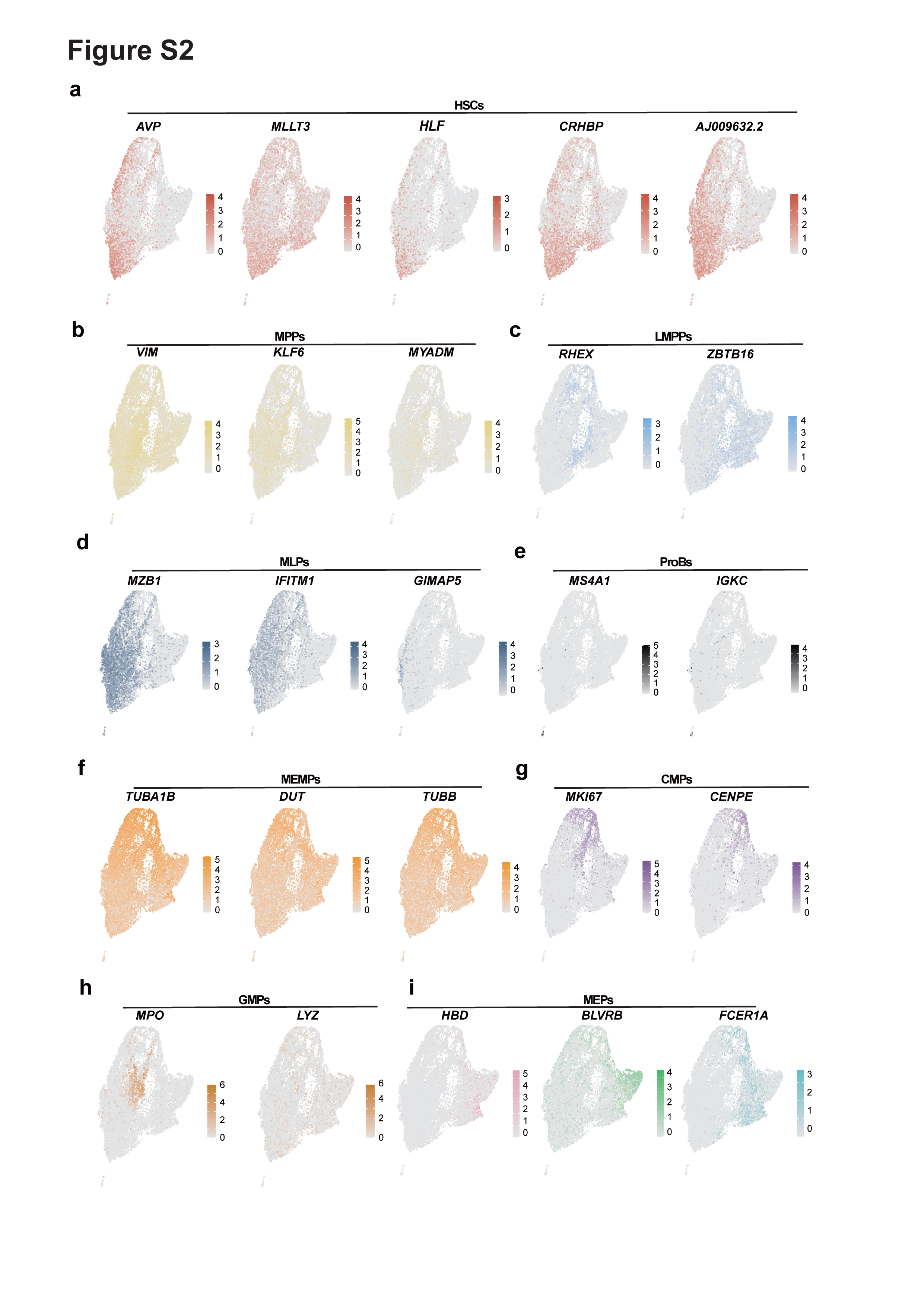

Supplement: Supplementary file 3 — Supporting Information [file CTM2-13-e1175-s001.png]

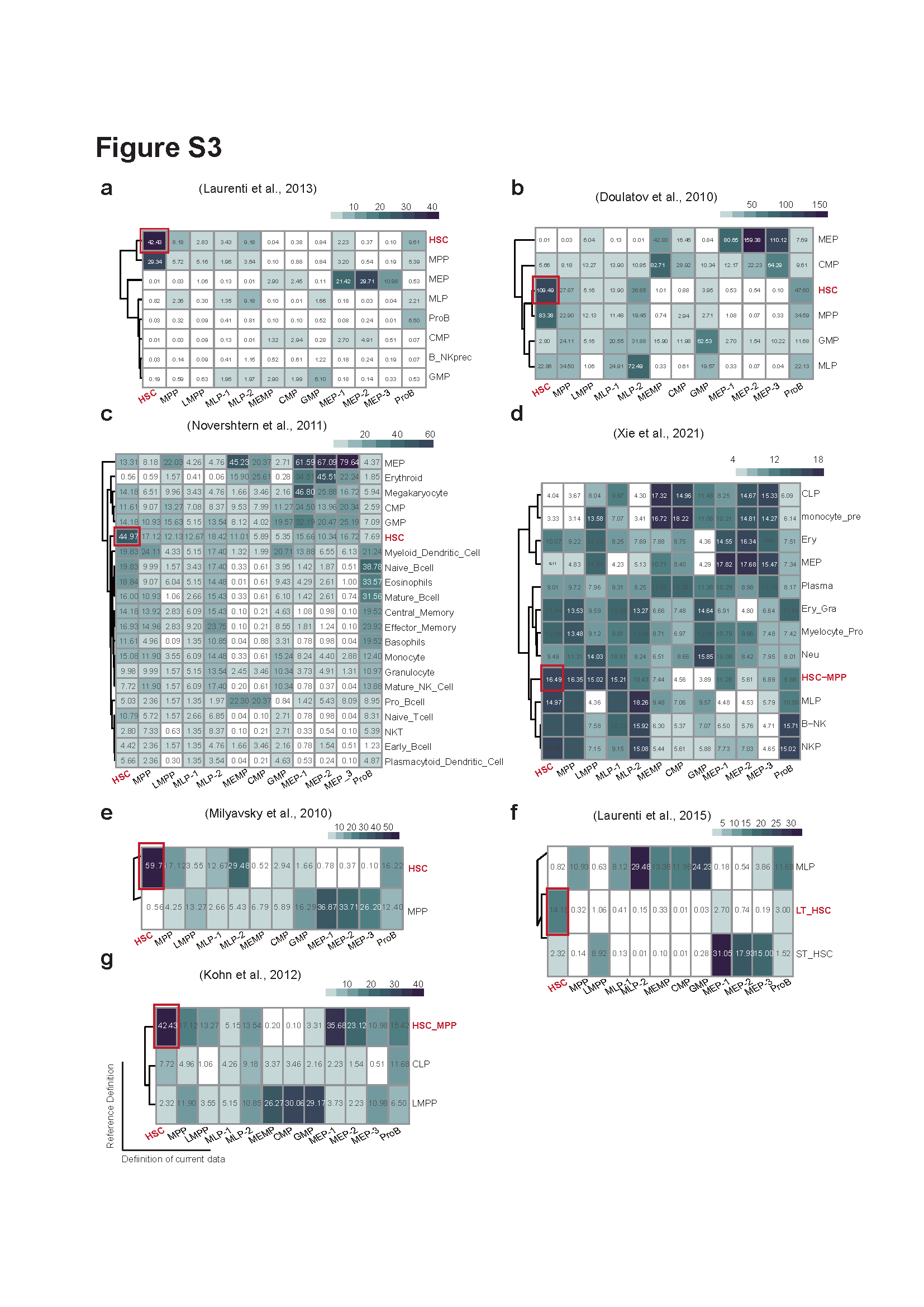

Supplement: Supplementary file 4 — Supporting Information [file CTM2-13-e1175-s007.png]

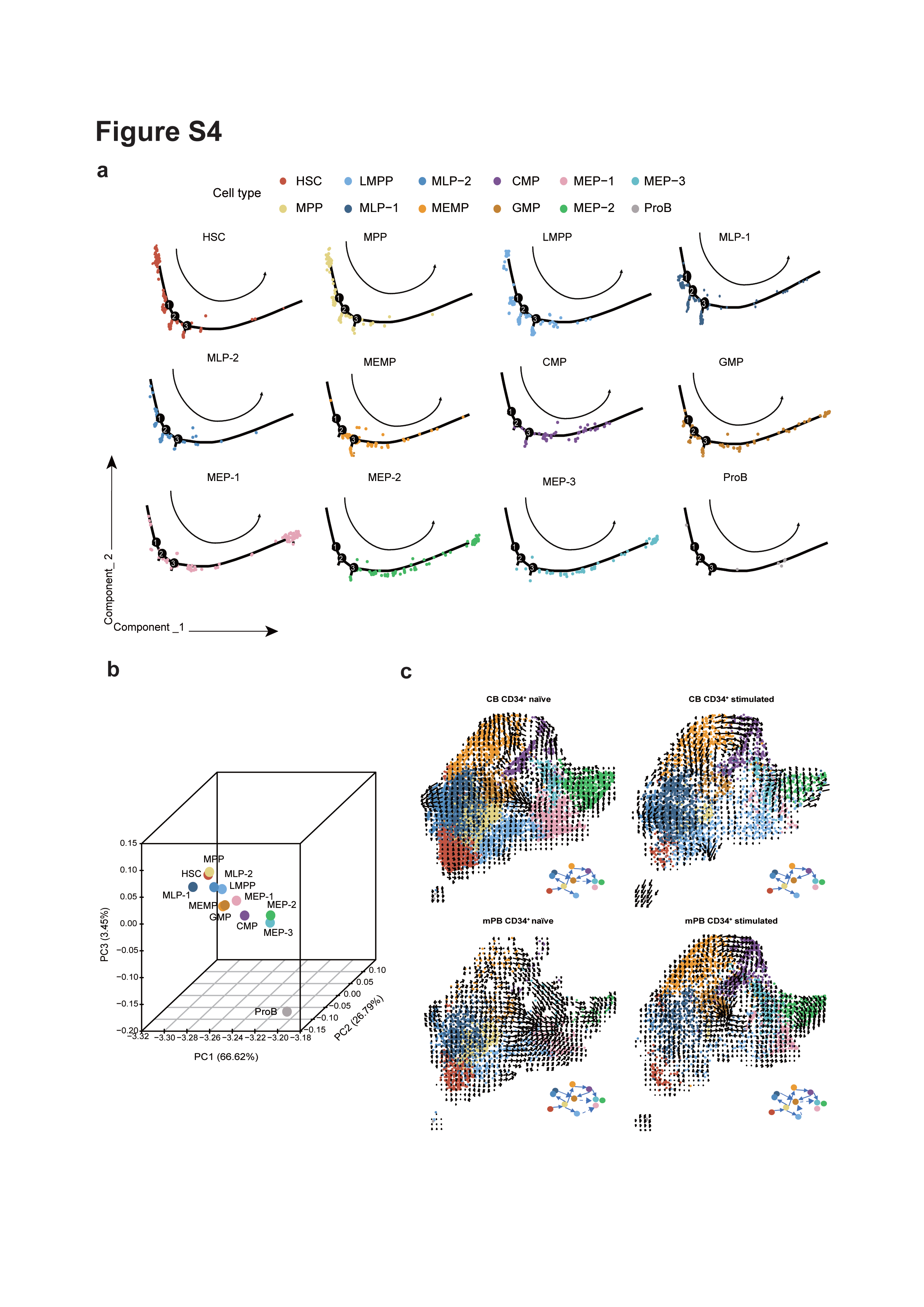

Supplement: Supplementary file 5 — Supporting Information [file CTM2-13-e1175-s003.png]

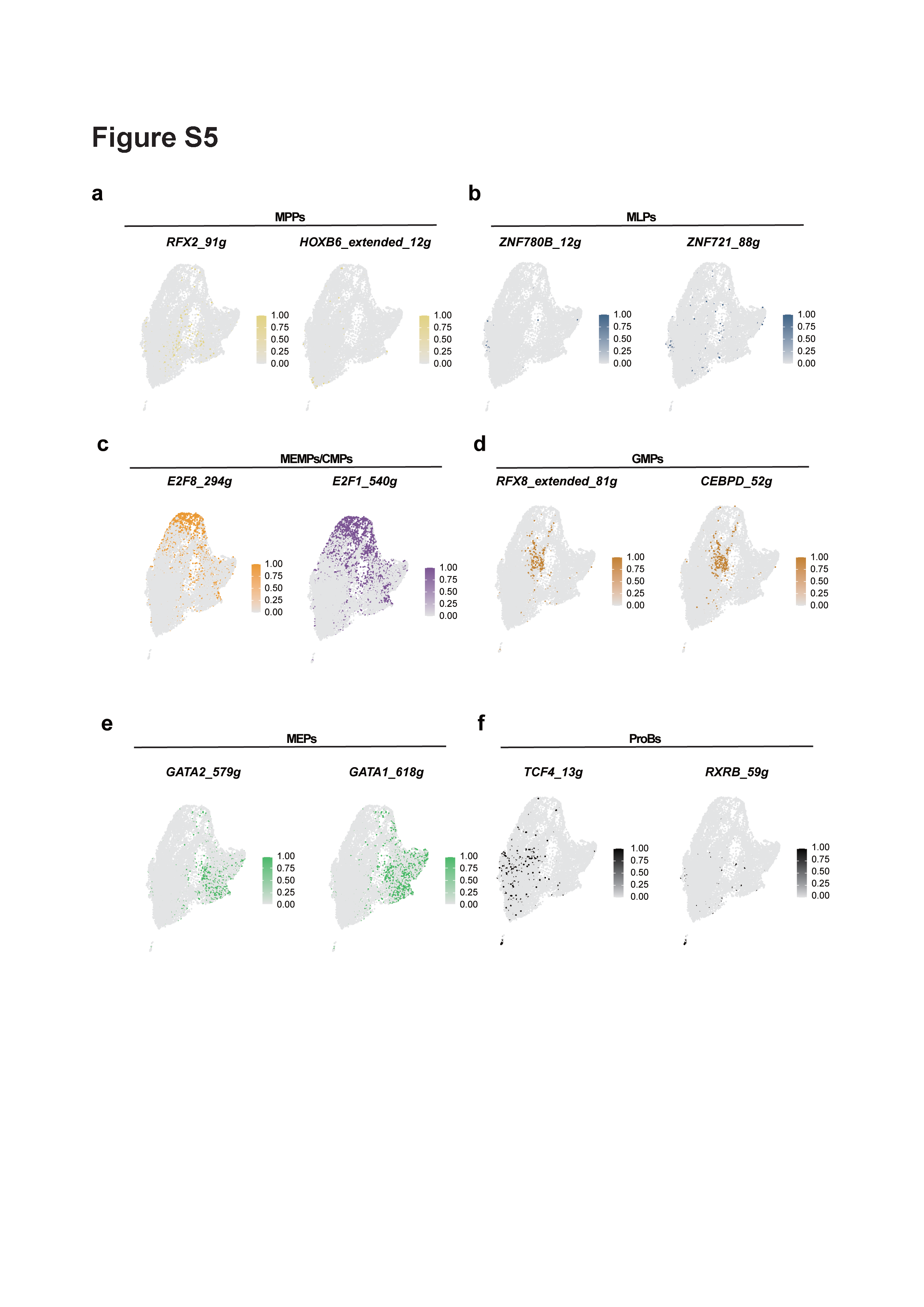

Supplement: Supplementary file 6 — Supporting Information [file CTM2-13-e1175-s002.png]

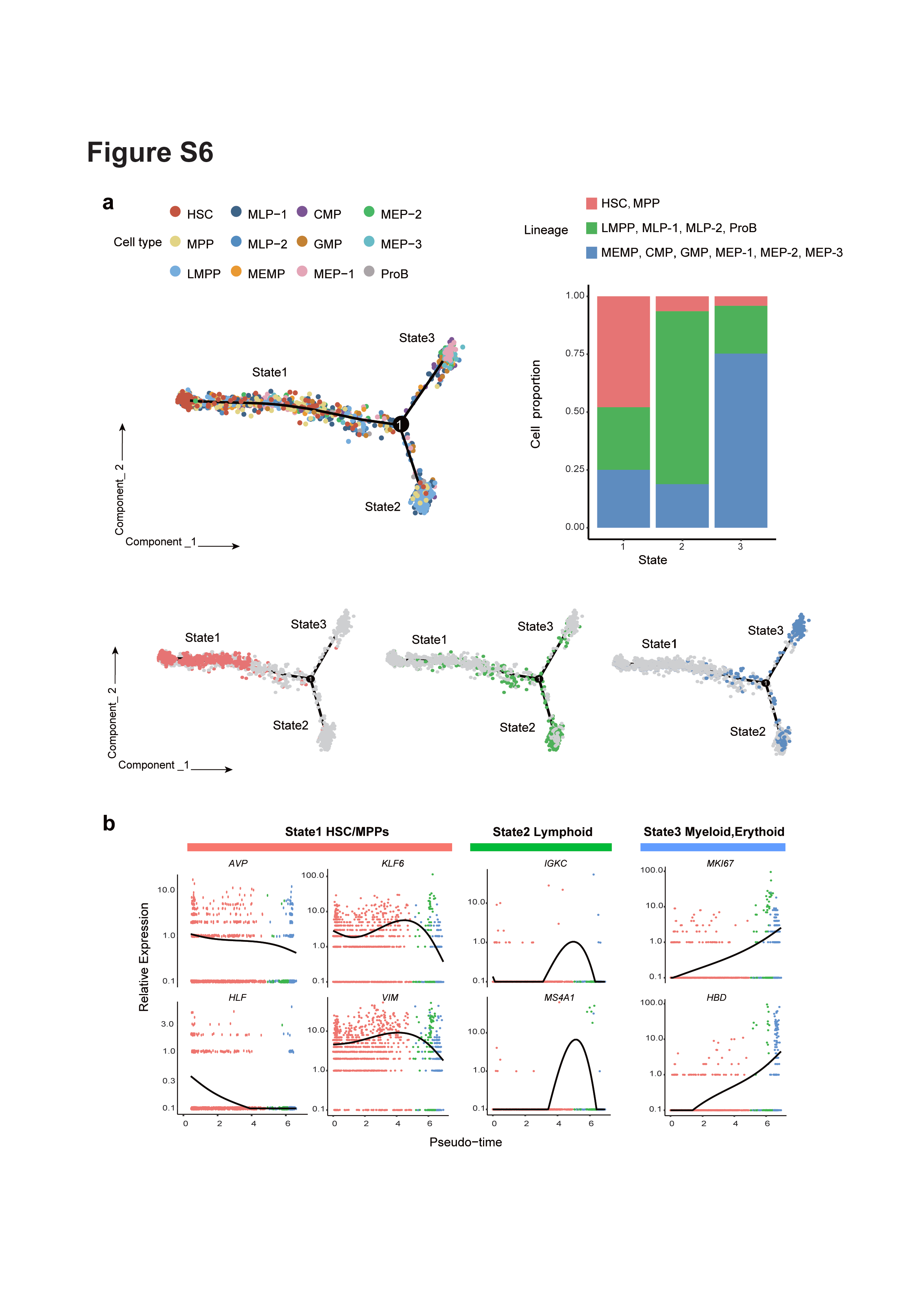

Supplement: Supplementary file 7 — Supporting Information [file CTM2-13-e1175-s005.png]
